# Bioinspired Reductionistic Peptide Engineering for Exceptional Mechanical Properties

**DOI:** 10.1038/srep16070

**Published:** 2015-11-03

**Authors:** M. B. Avinash, Devaraj Raut, Manish Kumar Mishra, Upadrasta Ramamurty, T. Govindaraju

**Affiliations:** 1 Bioorganic Chemistry Laboratory, New Chemistry Unit, Jawaharlal Nehru Centre for Advanced Scientific Research, Jakkur P. O., Bengaluru 560064, India; 2 Department of Materials Engineering, Indian Institute of Science, Bengaluru 560012, India; 3 Solid State and Structural Chemistry Unit, Indian Institute of Science, Bengaluru 560012, India; 4 Center of Excellence for Advanced Materials Research, King Abdulaziz University, Jeddah 21589, Saudi Arabia

## Abstract

A simple solution-processing and self-assembly approach that exploits the synergistic interactions between multiple hydrogen bonded networks and aromatic interactions was utilized to synthesize molecular crystals of cyclic dipeptides (CDPs), whose molecular weights (~0.2 kDa) are nearly three orders of magnitude smaller than that of natural structural proteins (50–300 kDa). Mechanical properties of these materials, measured using the nanoindentation technique, indicate that the stiffness and strength are comparable and sometimes better than those of natural fibres. The measured mechanical responses were rationalized by recourse to the crystallographic structural analysis and intermolecular interactions in the self-assembled single crystals. With this work we highlight the significance of developing small molecule based bioinspired design strategies to emulate biomechanical properties. A particular advantage of the successfully demonstrated reductionistic strategy of the present work is its amenability for realistic industrial scale manufacturing of designer biomaterials with desired mechanical properties.

Naturally occurring biological materials and structures in general are highly optimized to serve multifunctional purposes while being lightweight^1^,^2^,^3^. A key difference between these and man-made high performance structures are that the former are synthesized through ‘bottom-up’ approach using the combination of solvo-chemical and self-assembly routes^4^,^5^,^6^. Further, their remarkable mechanical performance is due to the ingenious use of weak noncovalent interactions^7^,^8^,^9^. Specifically, the mechanical properties of natural materials like cocoon and spider silks, and amyloid fibers can all be attributed to the exploitation of hydrogen bonding interactions. For example, theoretically predicted elastic modulus, *E*, of hydrogen bonded assemblies is ~10 GPa, which is effectively achieved in spider dragline silks^8^. It is in this context, we envision that it may be possible to synthesize biomolecular materials with desired mechanical properties by exploiting the crystal engineering principles. For this, we employ the simplest forms of peptide, *viz.* CDPs, which can be tailored into hydrogen bonded one-dimensional (1D) molecular chains or two-dimensional (2D) molecular layers depending on the nature of the side chains^10^,^11^,^12^,^13^,^14^,^15^,^16^. Such materials offer the following important advantages: (i) rigid structural and self-complementary (multiple hydrogen bonding) motifs, (ii) functional tailorability through the introduction of α-substituents to impart additional functionalities or noncovalent interactions, (iii) configurational variability, (iv) biocompatibility, (v) solution processability that entails easy synthesis and scalability.

In the current work, we employ CDPs of alanine (**LL-Ala** and **LD-Ala**) to exploit multiple hydrogen bonding interactions. Further, CDPs of an unnatural amino acid phenylglycine (**LL-Phg** and **LD-Phg**), with aromatic substituents were utilized to introduce additional noncovalent (aromatic) interactions (Fig. 1a–d)^17^,^18^,^19^,^20^. Here, LL and LD represent the stereochemistry of the two amino acids in CDP. This allows control over the spatial orientation of the amino acid side chain (α-substituent) and in turn modulation of the self-assembly of CDPs. It is important to note that the molecular weights of the CDPs utilized in the current study are only about 0.2 kDa, whereas the proteins in natural biomaterials such as silk and amyloid fibers are of few tens to few hundreds of kDa^21^. Such a reductionistic strategy was intentionally employed so as to simplify the molecular material’s design, which in turn would pave way for realistic industrial scale manufacturing, if the designed materials are found to have the desired properties.

## Results

**Figure 1:**
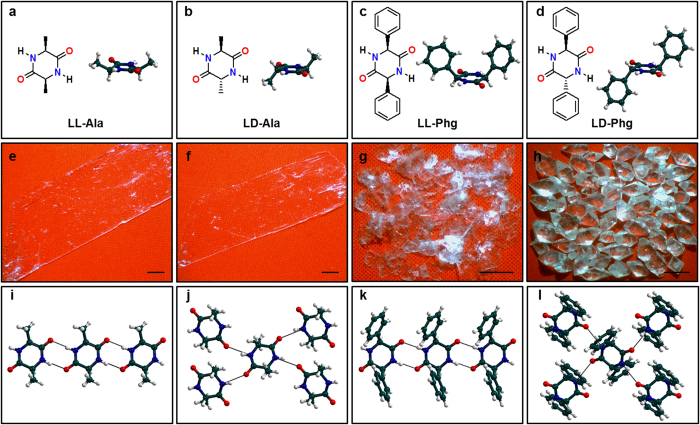
Molecular structures of CDPs and their organization in single-crystalline self-assembled architectures. Molecular structures of (**a**) **LL-Ala,** (**b**) **LD-Ala,** (**c**) **LL-Phg** and (**d), LD-Phg** along with their corresponding single-molecule crystal structure. Optical microscope images of self-assembled architectures of (**e**) **LL-Ala,** (**f**) **LD-Ala,** (**g**), **LL-Phg** and (**h**) **LD-Phg.** Scale bar 1 mm. Crystalline molecular packing of hydrogen bonded (**i**) 1D chains of **LL-Ala,** (**j**) 2D layers of **LD-Ala,** (**k**) 1D chains of **LL-Phg** and (**l**) 2D layers of **LD-Phg.** Hydrogen bonds are shown as dotted black lines.

Single-crystals of CDPs were obtained by self-assembly process in a solvent mixture comprising 50:50 (v/v) ratios of dichloromethane and methanol. **LL-Ala** and **LD-Ala** were obtained in the form of thin single-crystalline sheets that are 100 to 500 μm thick with 1 to 5 mm width and >10 mm length (Fig. 1e,f). **LL-Phg** was also obtained as single-crystal sheets with lateral dimensions of ~1 mm (Fig. 1g). **LD-Phg** formed rhombohedral shaped crystals that are several mm in size (Fig. 1h). All of these were subjected to single-crystal X-ray diffraction (XRD) studies in order to understand the molecular organization in them. **LL-Ala** sheets crystallized in the triclinic *P1* space group with the cyclic ring possessing non-planar conformation. Each **LL-Ala** molecule comprises two hydrogen bond donor (N-H) and acceptor (C = O) functionalities on either side of the CDP ring, which facilitate the formation of strong N-H···O hydrogen bond dimers with the two neighbouring molecules. These linkages, in turn, result in a 1D molecular chain that extends over the crystallographic *a* axis (Fig. 1i). The molecular organization in the **LD-Ala** crystals (monoclinic, *P21/n* space group), which were obtained by incorporating only one modification in the stereochemistry of the amino acid side chain of **LL-Ala**, are significantly different with a nearly-planar cyclic ring with the methyl functionalities on its either sides (Fig. 1b). This stereochemical modification facilitates the formation of four strong N-H···O hydrogen bonds with the neighbouring molecules for each **LD-Ala** molecule, which in turn results in the formation of 2D molecular layers as shown in Fig. 1j.

**Figure 2:**
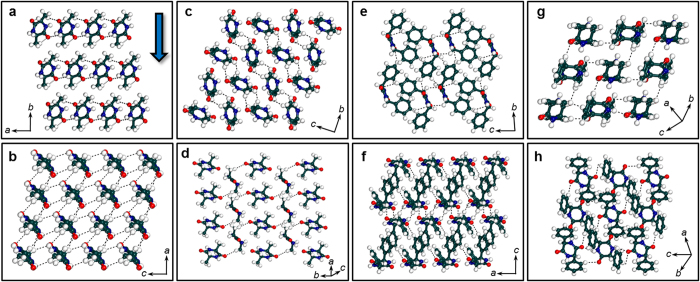
C-H···O interactions in CDPs. C-H···O interactions in (**a**,**b**) **LL-Ala**, (**c**,**d**) **LD-Ala**, (**e**,**f**) **LL-Phg**, and (**g**,**h**) **LD-Phg**. C-H···O interactions are shown as black dotted lines. (**a**,**c**,**e**,**g**) Molecular packing along the direction of indentation [as shown by blue arrow in **a**] and (**b**,**d**,**f**,**h**) molecular packing along within the plane of indentation [(010), (011), (010) and (11-1)] respectively.

In the crystals of **LL-Phg** that crystallize into triclinic *P*1 space group, each molecule binds to two neighboring molecules by means of strong N-H···O hydrogen bond dimers, which ultimately results in the formation of 1D molecular chains along the crystallographic *a* axis (Fig. 1k). In addition, the phenyl functionalities of **LL-Phg** were involved in π-π as well as CH-π interactions with the phenyl functionalities of neighbouring molecules along the crystallographic *a* axis (Supplementary Figure 1). **LD-Phg** was found to crystallize in orthorhombic *Pbca* space group to form rhombohedral architectures. Herein, each **LD-Phg** facilitates strong N-H···O hydrogen bonds as well as CH-π interactions with the four neighbouring molecules, which result in 2D molecular layers (Fig. 1l and Supplementary Figure 1). In summary, the LL stereochemistry of **LL-Ala** and **LL-Phg** favour the formation of 1D molecular chains, while the LD stereochemistry favours 2D molecular layers, as in **LD-Ala** and **LD-Phg**. In addition to the above discussed strong N-H···O hydrogen bonds, all four CDPs comprises of relatively weaker C-H···O interactions (Fig. 2 and Supplementary Table 4). **LL-Ala** showed the presence of 2D network of C-H···O interactions along the *ac* plane, while **LD-Ala** showed the presence of 1D chains (Fig. 2a–d). On the other hand, both **LL-Phg** and **LD-Phg** possess 2D networks of C-H···O interactions as shown in Fig. 2e–h. Therefore, with respect to C-H···O interactions, it is found that only **LD-Ala** possesses 1D chains whereas **LL-Ala**, **LL-Phg** and **LD-Phg** comprises of 2D networks.

**Figure 3:**
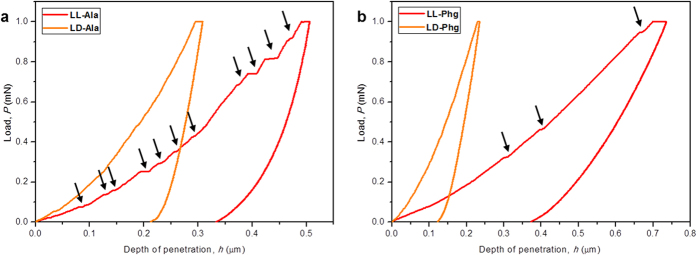
Nanoindentation studies of CDPs. Representative *P*-*h* curves of (**a**) **LL-Ala** & **LD-Ala** and (**b**) **LL-Phg** & **LD-Phg**.

Nanoindentation, which has been successfully employed to measure mechanical properties of organic and metal-organic framework crystals in the recent past, was utilized to evaluate the mechanical properties of the synthesized **LL-Ala**, **LD-Ala**, **LL-Phg** and **LD-Phg** crystals^22^,^23^,^24^,^25^,^26^,^27^,^28^,^29^,^30^,^31^. Representative load, *P*, vs. depth of penetration, *h*, curves obtained on the major faces of crystals, i.e. (010) of **LL-Ala,** (011) of **LD-Ala**, (010) of **LL-Phg** and (11-1) of **LD-Phg** are shown in Fig. 3a,b. While the loading segments of the *P*-*h* curves obtained on **LD-Ala** and **LD-Phg** are smooth, indicating to continuous plastic deformation, loading traces of **LL-Ala** exhibit several displacement bursts, which are often referred to as ‘pop-ins’ in the indentation literature, indicating to intermittent or jerky plastic flow^23^. On an average, 12 pop-ins were observed in case of **LL-Ala** and the average first pop-in load was found to be ~29 μN. Typically, the pop-in lengths, *h*_pop-in_, tend to be an integral multiple of relevant interplanar *d*-spacing of the crystal probed^23^. In the current context, they were ~4.10 nm, which is about five times *d*_*010*_ (=7.69 Å), suggesting that the pop-ins occur due to collective sliding of multiple (010) planes during indentation. Pop-ins were also noted on the loading segments of the *P*-*h* curves obtained on **LL-Phg**, but their number was much smaller. In this case, the average *h*_pop-in_ was ~4.75 nm, which again is close to five times the *d*_010_ (= 9.77 Å).

The *P*-*h* curves were analysed using the Oliver-Pharr method to extract elastic modulus, *E*, and hardness, *H*, of the crystals, which are listed in Table 1^23^. It is seen that the crystals with 2D hydrogen bond network (**LD-Ala** and **LD-Phg**) have far superior mechanical properties as compared to those with 1D hydrogen bonded chains (**LL-Ala** and **LL-Phg**). Amongst the four materials examined, **LD-Phg** is mechanically the most robust, with highest values of *E* and *H*. Notably, it is nearly-ten times stiffer and five times harder than **LL-Phg**. Likewise, *E* and *H* of **LD-Ala** are both more than double the respective values of **LL-Ala**. The measured *E* value of **LD-Ala**, which only contains hydrogen bonded assemblies, is in the range of 10–20 GPa predicted for such structures^8^. The synergistic interactions between hydrogen bonded networks and the aromatic interactions in **LD-Phg** lead to doubling of *E* in it *vis-á-vis* that of **LD-Ala**.

**Figure 4:**
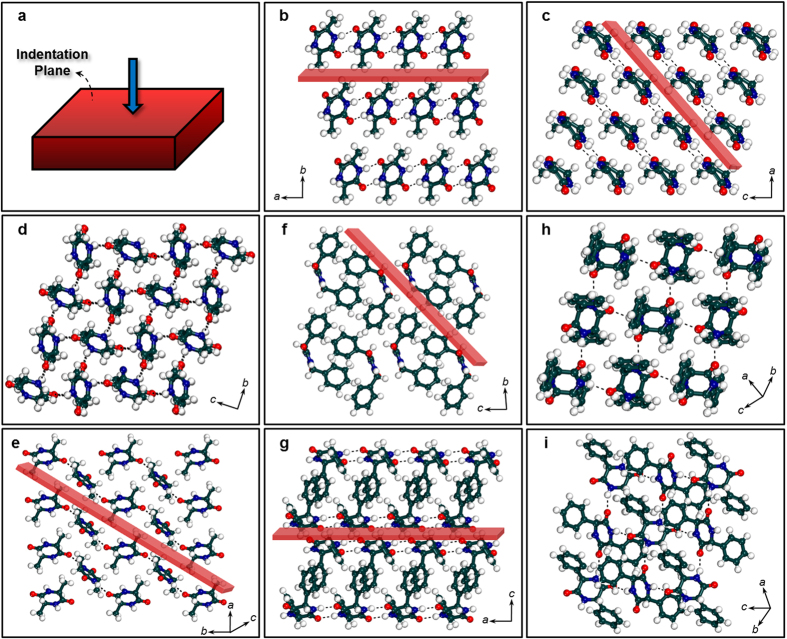
Molecular organization of CDPs along the direction of indentation and within the indentation plane. (**a**) Schematic of a typical crystal showing the plane (in red) on which indentations were made with respect to the crystal packing. Crystalline molecular packing of (**b**,**c**) **LL-Ala**; (**d**,**e**) **LD-Ala**; (**f**,**g**) **LL-Phg** and (**h**,**i**) **LD-Phg**. (**b**,**d**,**f**,**h**) Molecular packing along the direction of indentation [same as shown in (**a**) i.e. vertical] and (**c**,**e**,**g**,**i**) molecular packing along within the plane of indentation [(010), (011), (010) and (11-1)] respectively. Hydrogen bonds are shown as dotted black lines. Red plates in (**b**,**c**,**e**,**f**,**g**) show the slip planes. For (**d**) the slip plane is parallel to the indentation direction and for (**h**,**i**) there are no clear slip planes due to their interconnected network.

Structural reasons for such large differences in mechanical responses of the CDPs were sought through the examination of crystal packings (Fig. 4). In case of **LL-Ala**, strong N-H···O [*D* = distance between N and O in N-H···O; *d* = distance between H and O in N-H···O; *θ* = bond angle of N-H···O: 2.89 Å; 1.89 Å; 170°] hydrogen bonded 1D chains run parallel to the (010) indentation plane (Fig. 4b). These chains, which are aligned perpendicular to the indentation axis, are interlinked to each other by weak van der Waals interactions along the (010) plane (Fig. 4c). In contrast, **LD-Ala** comprises of strong N-H···O (2.88 Å; 1.93 Å; 157°) hydrogen bonded 2D layers that are oriented parallel to the indentation direction as shown in Fig. 4d,e. Herein, it should also be noted that **LL-Ala** and **LD-Ala** consists of 2D network and1D chains of C-H···O interactions, respectively. Thus, the interlocked and corrugated 2D molecular packing of strong N-H···O interactions in **LD-Ala** imparts structural rigidity to the crystal, which in turn manifests in terms of high *E* and *H*. In comparison, **LL-Ala**, which consists of only stacks of 1D chains of N-H···O interactions, is relatively compliant and softer. Therefore, for higher *E* and *H*, it is the nature and the strength of N-H···O interactions which contributes greatly over that of weaker C-H···O interactions, as observed in case of **LL-Ala** and **LD-Ala**. While the crystal structure of **LL-Phg** also contains strong N-H···O (2.88 Å; 1.87 Å; 172°) hydrogen bonded 1D chains (Fig. 4f,g), they facilitate additional π-π and C-H···π interactions that are oblique to the indentation direction. Similarly, the (11-1) indentation face of **LD-Phg** encompasses strong N-H···O (2.95 Å; 2.00 Å; 150°) hydrogen bonded 2D networks that are oriented skew to both the indentation plane and the direction of indentation (Fig. 4h,i). The presence of strong hydrogen bonded 2D networks as well as additional synergistic contributions from intermolecular C-H···π interactions make **LD-Phg** stiffer and stronger than the alanine derivatives (**LL-Ala** and **LD-Ala**). The relatively lower *H* of **LL-Phg**, *vis-á-vis* **LD-Phg** can be ascribed to the presence of molecular slip planes along *a*-axis that shear slide relatively easily and smoothly during indentation. Thus, **LD-Phg** and **LL-Phg** respectively represent the synergistic and non-synergistic contributions of additional aromatic interactions that modulate *E* and *H* of hydrogen bonded organic materials. Note that both **LL-Ala** and **LL-Phg** contain relatively stronger hydrogen bonds (*θ* = ~170°) in comparison to their LD counterparts (*θ* of **LD-Ala** = 157°, *θ* of **LD-Phg** = 150°). Yet, the LD derivatives exhibit superior mechanical properties as compared to their LL counterparts. This observation suggests that the mechanical behavior of these biomolecular materials depend strongly on the hydrogen bond networks as well as synergistic contributions from other noncovalent interactions (as in case of **LD-Phg**) and is not exclusively dependent on the strength of N-H···O interactions.

## Discussion

**Figure 5:**
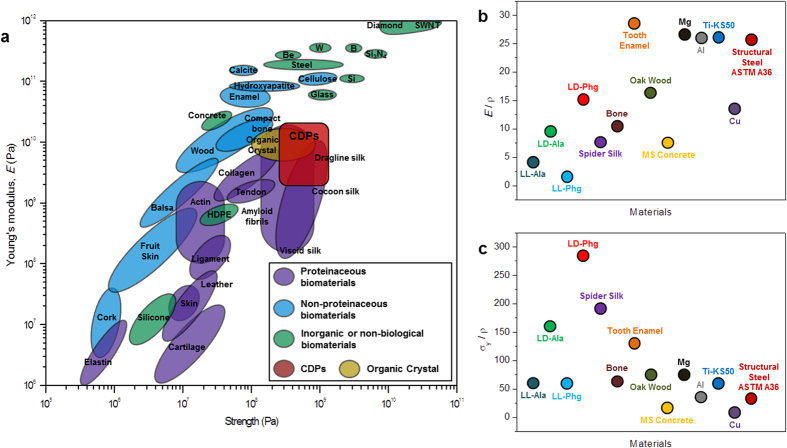
Mechanical properties of CDPs and other materials. (**a**) Plot of elastic modulus (*E*) verses strength for various materials and CDPs. Adapted from ref. 8. Specific properties of CDPs and other materials obtained by plotting (**b**) ratio of *E* to density and (**c**) ratio of yield strength (σ_y_) to density. Organic Crystal: Compounds enlisted in Supplementary Table 1. HDPE: High-density polyethylene; SWNT: Single-wall carbon nanotube; MS Concrete: Mild-strength concrete; Ti-KS50: Titanium-KS50.

The properties obtained on the bioorganic crystals synthesized in this work are put in perspective through a comparison of nanoindentation data available on various organic crystals (Supplementary Table 1). It indicates that **LD-Phg** is by far the stiffest and hardest organic crystal amongst those that have been examined hitherto (Fig. 5a). Further, its *E* is comparable in fact slightly higher than the *E* of 19 GPa that was estimated through computational studies for diphenylalanine based nanotubes^32^. Moreover, **LD-Phg** possesses very high yield strength, σ_y_ (estimated using the relation σ_y_ = *H*/3) of 388 MPa^33^. In a broader context, it is worth noting that **LD-Phg**’s specific properties (ratios of *E* and σ_y_ to density) are comparable to the respective values of structural metals (Supplementary Table 2) as it has low density of ~1.3 gcm^−3^ (Fig. 5b,c).

In conclusion, the work presented in this paper demonstrates that it is possible to design peptide-based organic materials that are as strong and stiff as some of the best known natural fibres. This bioinspired design strategy employs a reductionistic method and exploits the synergistic interactions between hydrogen bonded networks and aromatic interactions in the self-assembled molecular architectures. Further, the substantial differences in the mechanical responses of the different CDP crystals demonstrate that it is possible to design bioinspired organic materials with tuneable mechanical properties, on the basis of molecular crystal engineering principles. Additionally, such low-density and high-strength biomolecular materials offer the advantages of biocompatibility, solution processability and large-scalability, and thus are promising in the contexts of biomaterial applications.

## Methods

### Materials

All the solvents and reagents were obtained from Sigma-Aldrich and used as received unless otherwise mentioned.

### NMR Spectroscopy, Mass Spectrometry (HRMS), and Elemental Analysis

^1^H and ^13^C NMR were recorded on a Bruker AV-400 spectrometer with chemical shifts reported as ppm (in CDCl_3_ with tetramethylsilane as internal standard). High resolution mass spectra (HRMS) were obtained on Agilent Technologies 6538 UHD Accurate-Mass Q-TOF LC/MS spectrometer. Elemental analysis was carried out on ThermoScientific FLASH 2000 Organic Element Analyzer.

### Optical Microscopy

Optical images of macroscopic architectures of **LL-Ala**, **LD-Ala**, **LL-Phg** and **LD-Phg** were acquired with a Motic upright microscope attached to a CCD camera from Suntech technologies.

### Single-crystal X-ray diffraction

Single-crystalline macroscopic architectures of **LL-Ala**, **LD-Ala**, **LL-Phg** and **LD-Phg** were obtained by self-assembly in 50:50 (v/v) compositions of dichloromethane and methanol. X-ray diffraction studies were carried out on a Rigaku Mercury 375R/M CCD (XtaLAB mini) diffractometer using graphite monochromatic Mo Kα radiation (λ = 0.7 Å) attached with a Rigaku low-temperature gas spray cooler. The cell parameters obtained for the crystalline forms of **LL-Ala**, **LD-Ala** and **LD-Phg** were found to be same as that of reported structure in the CSD (version 5.35, www.ccdc.cam.ac.uk). On the other hand, the crystallographic data of **LL-Phg** was processed with the Rigaku Crystal Clear software^34^. Structure solution and refinement were carried out using SHELX97^35^ incorporated in the WinGXsuite^36^. Face indexing of good quality single crystals of all CDPs were performed with Crystal Clear software, and the major faces were assigned accordingly.

### Nanoindentation

Nanoindentation studies are performed on the samples using the Triboindenter (Hysitron, Minneapolis, USA) with *in-situ* imaging capability. The machine continuously monitors the load, *P*, and the depth of the penetration, *h*, of the indenter with the resolutions of 1 nN and 0.2 nm, respectively. A Berkovich diamond tip indenter with the tip radius of ~100 nm is used for the indentation. A peak load, *P*_*max*_ of 1 mN with the loading and unloading rates of 0.2 mN s^−1^ and a hold time (at *P*_*max*_) of 5 s is employed. A minimum of 50 indentations are performed in each case and the average is reported. The *P*-*h* curves were analyzed using the Oliver-Pharr method to extract the elastic modulus (*E*), and the hardness (*H*) of the samples.

### Synthesis of LL-Ala

9-Fluorenylmethoxycarbonyl protected L-alanine (Fmoc-L-Ala-OH) and L-alanine methylester (L-Ala-OMe) were prepared by using standard protection protocols. Fmoc-L-Ala-OH (2.24 g, 7.2 mmol) dissolved in dichloromethane was added with L-Ala-OMe (1 g, 7.2 mmol), 1-ethyl-3-(3-dimethylaminopropyl)carbodiimide (EDC.HCl, 1.65 g, 8.64 mmol) and 1-hydroxybenzotriazole (HOBt, 1.17 g, 8.64 mmol). The solution was maintained at ice cold temperature. Diisopropylethylamine (DIPEA, 3.26 g, 25.2 mmol) was added and the reaction mixture was stirred at ice temperature for 1 h and then at room temperature for 5 h. The reaction progress was monitored by thin layer chromatography (TLC). Reaction mixture was evaporated to dryness and extracted from dichloromethane, washed with water, dried over anhydrous sodium sulphate. The solvent was evaporated to obtain Fmoc-L-Ala-L-Ala-OMe in quantitative yield. The Fmoc-deprotection of Fmoc-L-Ala-L-Ala-OMe dipeptide in 15% piperidine/dichloromethane resulted in intramolecular cyclization to give **LL-Ala**, which was filtered, washed with dichloromethane, methanol and the crude white solid material was further recrystallized to obtain **LL-Ala**. ^1^H NMR (*CDCl*_*3*_*-CF*_*3*_*COOH*, 400 MHz, *δ*) 8.27 (s, 2H, NH), 4.32 (q, *J* = 7.2 Hz, 2H, CH), 1.62 (d, *J* = 6.8 Hz, 6H, CH_3_); ^13^C NMR (*CDCl*_*3*_*-CF*_*3*_*COOH*, 100 MHz, *δ*) 171.6, 50.9, 19.7; HRMS (ESI-MS): *m*/*z* = 143.0808 [M + H]^+^ for C_6_H_11_N_2_O_2_ (calc. 143.0815); Elemental analysis: Calcd. for C_6_H_10_N_2_O_2_: C, 50.69; H, 7.09; N, 19.71. Found: C, 50.65; H, 7.14; N, 19.69.

### Synthesis of LD-Ala

9-Fluorenylmethoxycarbonyl protected L-alanine (Fmoc-L-Ala-OH) and D-alanine methylester (D-Ala-OMe) were prepared by using standard protection protocols. Fmoc-L-Ala-OH (2.24 g, 7.2 mmol) dissolved in dichloromethane was added with D-Ala-OMe (1 g, 7.2 mmol), 1-ethyl-3-(3-dimethylaminopropyl)carbodiimide (EDC.HCl, 1.65 g, 8.64 mmol) and 1-hydroxybenzotriazole (HOBt, 1.17 g, 8.64 mmol). The solution was maintained at ice cold temperature. Diisopropylethylamine (DIPEA, 3.26 g, 25.2 mmol) was added and the reaction mixture was stirred at ice temperature for 1 h and then at room temperature for 5 h. The reaction progress was monitored by thin layer chromatography (TLC). Reaction mixture was evaporated to dryness and extracted from dichloromethane, washed with water, dried over anhydrous sodium sulphate. The solvent was evaporated to obtain Fmoc-L-Ala-D-Ala-OMe in quantitative yield. The Fmoc-deprotection of Fmoc-L-Ala-D-Ala-OMe dipeptide in 15% piperidine/dichloromethane resulted in intramolecular cyclization to give **LD-Ala**, which was filtered, washed with dichloromethane, methanol and the white solid material was further recrystallized to obtain **LD-Ala**. ^1^H NMR (CDCl_3_-CF_3_COOH, 400 MHz, *δ*) 8.29 (s, 2H, NH), 4.31 (q, *J* = 7.2 Hz, 2H, CH), 1.62 (d, *J* = 6.8 Hz, 6H, CH_3_); ^13^C NMR (CDCl_3_-CF_3_COOH, 100 MHz, *δ*) 171.6, 50.9, 19.7; HRMS (ESI-MS): *m*/*z* = 143.0810 [M + H]^+^ for C_6_H_11_N_2_O_2_ (calc. 143.0815); Elemental analysis: Calcd. for C_6_H_10_N_2_O_2_: C, 50.69; H, 7.09; N, 19.71. Found: C, 50.67; H, 7.15; N, 19.67.

### Synthesis of LL-Phg & LD-Phg

These were synthesized as per our earlier report^12^.

## Additional Information

**How to cite this article**: Avinash, M. B. *et al.* Bioinspired Reductionistic Peptide Engineering for Exceptional Mechanical Properties. *Sci. Rep.* **5**, 16070; doi: 10.1038/srep16070 (2015).

## Supplementary Material

Supplementary Information

## Figures and Tables

**Table 1:** Elastic modulus, hardness and other parameters of CDPs.

Material	Crystal system	Space group	Hydrogen bond network	Hydrogen bond distance (Å)	Hydrogen bond angle (°)	Indented face	Slip plane Slip direction	*E* (GPa)	*H* (MPa)
LL-Ala	Triclinic	*P*1	1D	2.89 (1.89)	170	{010}	{100} [100]	5.4 ± 0.76	240.5 ± 23
LD-Ala	Monoclinic	*P*2_1_*/n*	2D	2.88 (1.93)	157	{011}	{001} [110]	12.6 ± 1.44	642.1 ± 116
LL-Phg	Triclinic	*P*1	1D	2.88 (1.87)	172	{010}	{011} [10-1]	2.13 ± 0.16	240.3 ± 78
LD-Phg	Orthorhombic	*Pbca*	2D	2.95 (2.00)	150	{11-1}	{100} [200]	20.5 ± 0.66	1163.9 ± 75
