# Supplementary material for: Bioinspired Reductionistic Peptide Engineering for Exceptional Mechanical Properties
Source: Sci Rep. 2015 Nov 3;5:16070. doi: 10.1038/srep16070 (PMC4630637; doi:10.1038/srep16070)
Supplement: Supplementary Information [file srep16070-s1.pdf]

Bioinspired Reductionistic Peptide Engineering for Exceptional Mechanical Properties

M. B. Avinash[1], Devaraj Raut[2], Manish Kumar Mishra[3], Upadrasta Ramamurty[2,4*], and T. Govindaraju[1*]

[1]Bioorganic Chemistry Laboratory, New Chemistry Unit, Jawaharlal Nehru Centre for Advanced Scientific Research, Jakkur P. O., Bengaluru 560064, India.

[2]Department of Materials Engineering, Indian Institute of Science, Bengaluru 560012, India.

[3]Solid State and Structural Chemistry Unit, Indian Institute of Science, Bengaluru 560012, India.

[4]Center of Excellence for Advanced Materials Research, King Abdulaziz University, Jeddah 21589, Saudi Arabia

*e-mail: ramu@materials.iisc.ernet.in; tgraju@jncasr.ac.in

Contents

1. Aromatic interactions in **LL-Phg** and **LD-Phg**
2. Optical microscope image of **LL-Phg** and **LD-Phg**
3. Atomic force microscopy (AFM) images of nanoindents
4. Pop-in analysis data of **LL-Ala**
5. Pop-in analysis data of **LL-Phg**
6. Face indexing of **LL-Ala**
7. Face indexing of **LD-Ala**
8. Face indexing of **LL-Phg**
9. Face indexing of **LD-Phg**
10. List of elastic modulus and hardness of organic crystals
11. List of mechanical properties of CDPs and other materials
12. Crystallographic information table of CDPs
13. List of hydrogen bonds and related parameters in crystals of CDPs
14. References

1. Aromatic interactions in LL-Phg and LD-Phg

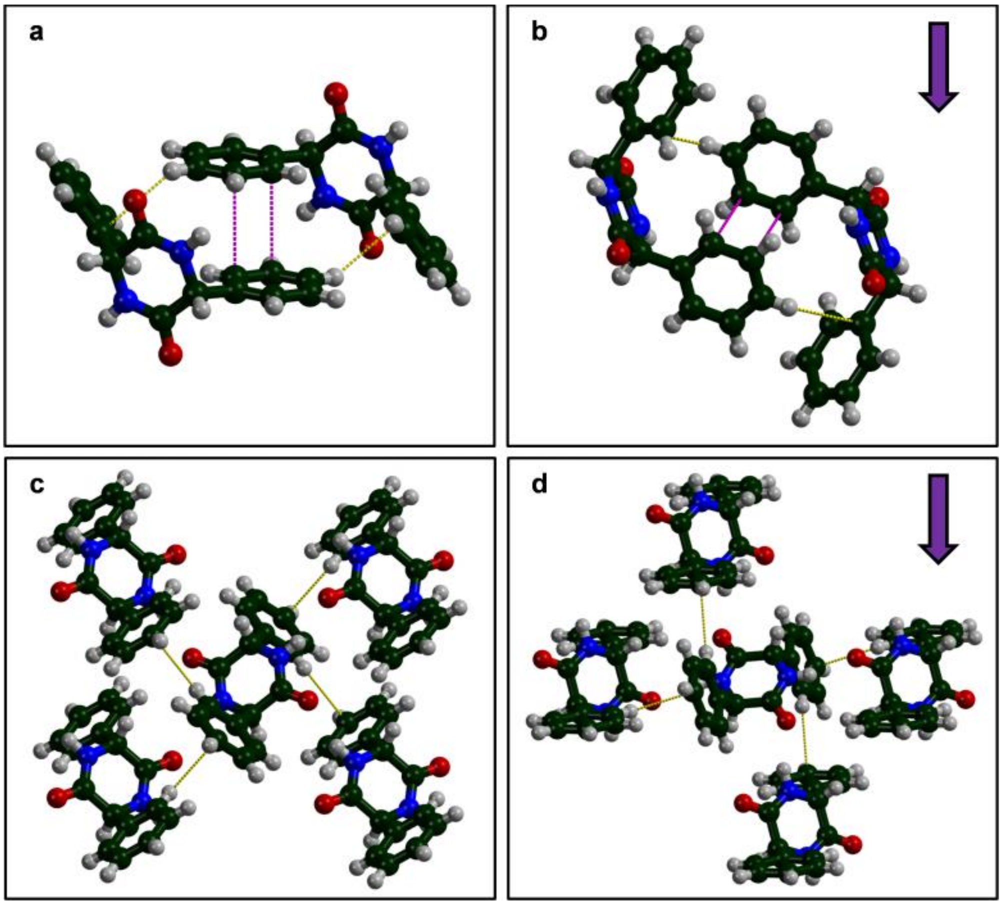

Figure 1. Aromatic interactions in **a**, **b**, **LL-Phg** and **c**, **d**, **LD-Phg**. CH-π and π-π interactions are shown as yellow and magenta dotted lines. The arrows in **b**, **d**, show the direction of indentation.

2. Optical microscope images of LL-Phg and LD-Phg

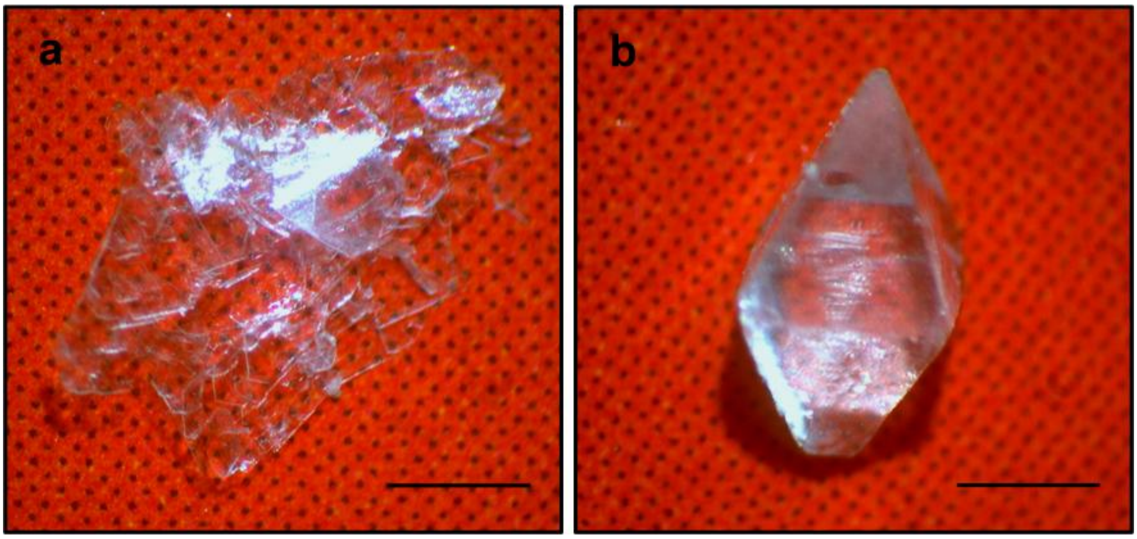

Figure 2: Optical microscope images of **a**, **LL-Phg** and **b**, **LD-Phg**. Scale bar 0.5 mm.

3. Atomic force microscopy (AFM) images of nanoindents

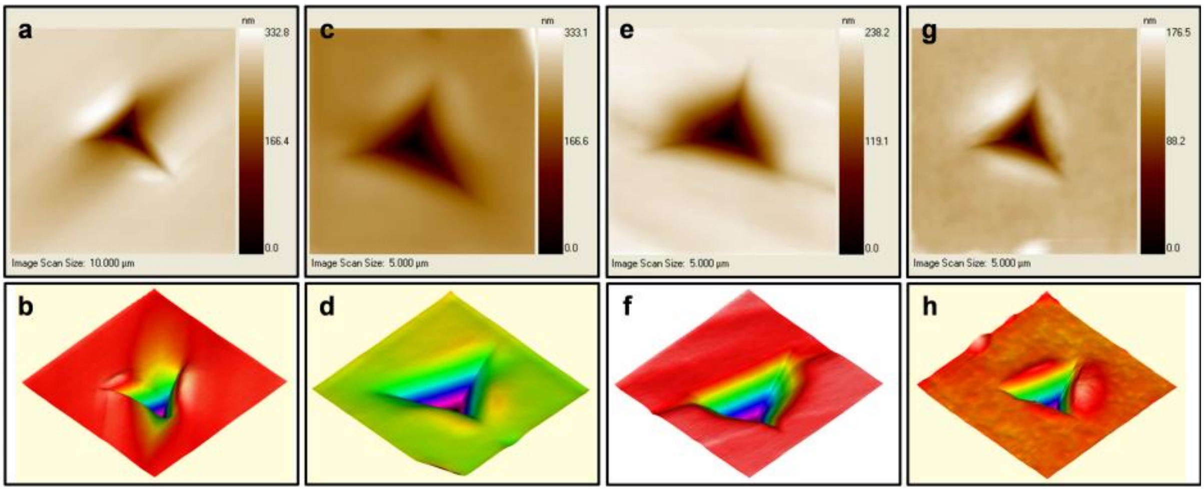

Figure 3: Atomic force microscopy (AFM) images of nanoindents performed on **a**, **b**, (010) face of **LL-Ala**; **c**, **d**, (011) face of **LD-Ala**; **e**, **f**, (010) face of **LL-Phg** and **g**, **h**, (11-1) face of **LD-Phg**. **b**, **d**, **f**, **h**, The three-dimensional AFM image of corresponding CDP.

4. Pop-in analysis data of LL-Ala

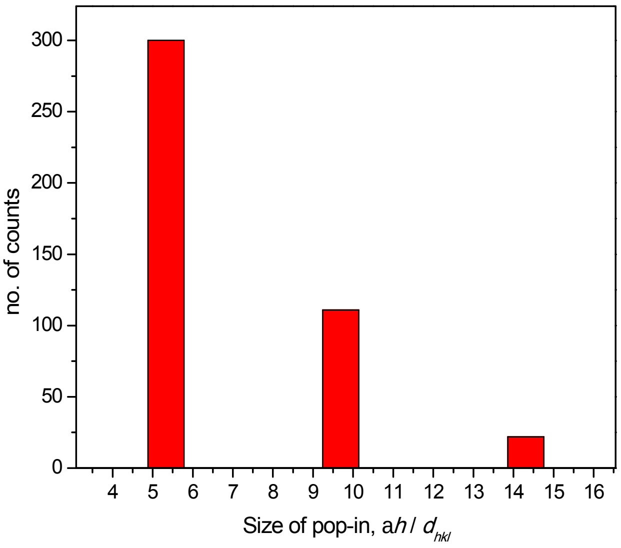

Figure 4: Pop-in analysis data of **LL-Ala**

5. Pop-in analysis data of LL-Phg

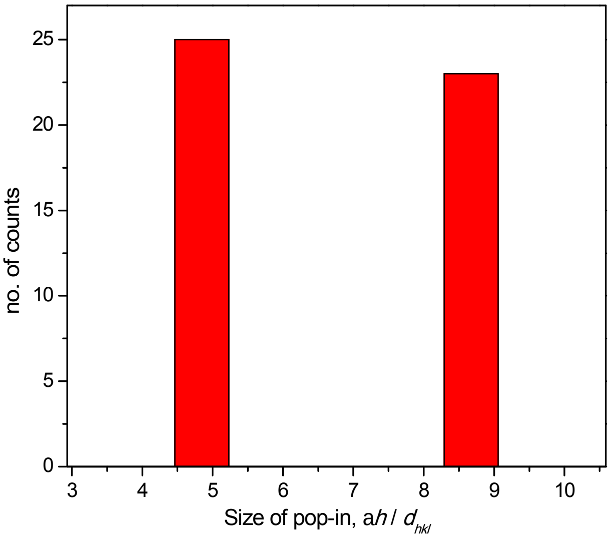

Figure 5: Pop-in analysis data of **LL-Phg**

6. Face indexing of LL-Ala

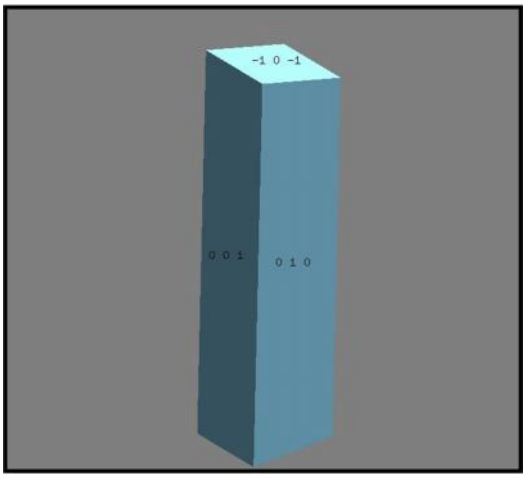

Figure 6: Image shows the face indexing of **LL-Ala** crystal.

7. Face indexing of LD-Ala

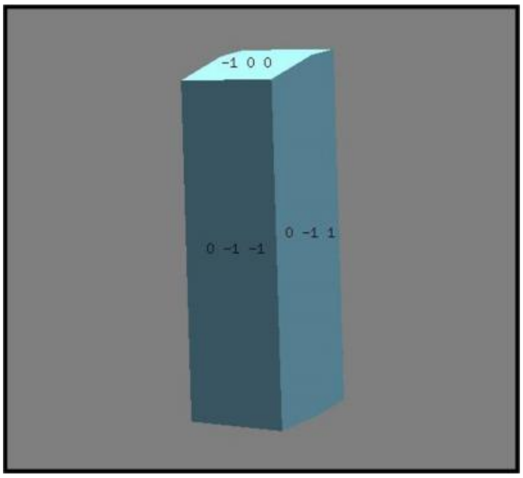

Figure 7: Image shows the face indexing of **LD-Ala** crystal.

8. Face indexing of LL-Phg

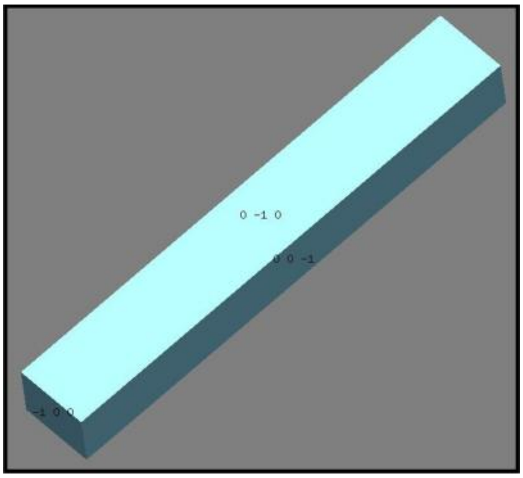

Figure 8: Image shows the face indexing of **LL-Phg** crystal.

9. Face indexing of LD-Phg

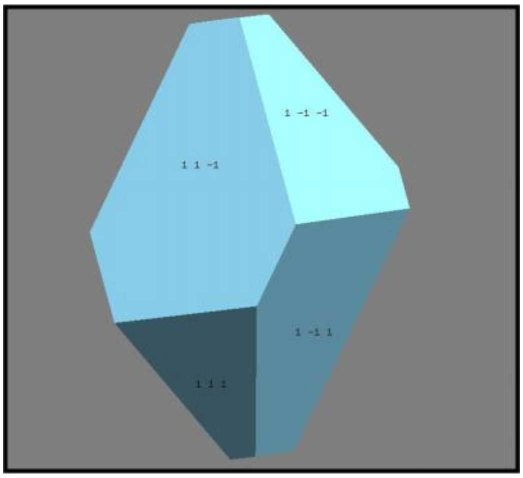

Figure 9: Image shows the face indexing of **LD-Phg** crystal.

10. List of elastic modulus and hardness of organic crystals (Table 1)

Compounds	*E* (GPa)	*H* (GPa)
Saccharin[1]	13.36 ± 0.05	0.610 ± 0.01
Aspirin[2]	9.57 ± 0.20	0.240 ± 0.008
(1,2,4,5-Tetracyanobenzene)-Pyrene co-crystal[3]	4.98 ± 0.24	0.143 ± 0.01
(1,2,4,5-Tetracyanobenzene)-Phenanthrene co-crystal[3]	5.76 ± 0.115	0.140 ± 0.001
Caffeine - (4-chloro-3-nitrobenzoic acid) co-crystal[4]	7.86 ± 0.08	0.175 ± 0.005
Difluoroboron Avobenzone[5]	13.2 ± 0.16	0.410 ± 0.011
1,2,4,5-Tetrabromobenzene[6]	6.41 ± 0.12	0.672 ± 0.009
Curcumin[7]	11.15 ± 0.20	0.432 ± 0.016
Sulfathiazole[7]	18.47 ± 0.36	0.851 ± 0.030
β-Piroxicam[8]	13.09 ± 0.22	0.53 ± 0.20
Felodipine[9]	12.81 ± 0.19	0.553 ± 0.016
Malonic acid[10]	9.13 ± 0.60	-
Succinic acid[10]	14.01 ± 0.75	-
Glutaric acid[10]	7.28 ± 0.90	-
Adipic acid[10]	11.53 ± 0.49	-
Pimelic acid[10]	8.23 ± 0.85	-
Suberic acid[10]	9.64 ± 0.39	-
Azelaic acid[10]	8.28 ± 0.38	-

The above mentioned *E* and *H* values of sulfathiazole were obtained for a peak load of 1 mN and not for 6 mN, as reported in ref. 7.

11. List of mechanical properties of spider silk and other materials (Table 2)

Material	Density, ρ gcm^{-3}	Elastic Modulus, E (GPa)	E / ρ	Yield Strength, σ_y (MPa)	σ_y/ρ
**LL-Ala**	1.31	5.4	4.1	80	61
**LD-Ala**	1.33	12.6	9.5	214	161
**LL-Phg**	1.33	2.1	1.6	80	60
**LD-Phg**	1.36	20.5	15.1	388	285
Spider silk[11]	1.31	10	7.6	250	191
Bone[11]	1.9	20	10.5	120	63
Tooth enamel[12,13]	2.9	83	28.6	380[a]	131
Red Oak Wood[12,13]	0.8	13	16.3	60[a]	75
Medium-Strength Concrete[12,13]	2.4	18	7.5	40[a]	17
Magnesium[14]	1.7	45	26.5	130	76
Aluminium[14]	2.7	70	25.9	95	35
Titanium KS50 [15]	4.5	116	25.8	270	60
Copper[14]	8.9	120	13.5	70	8
Structural Steel ASTM A36 [14]	7.8	200	25.6	250	32

[a]Compressive strength

12. Crystallographic information table of CDPs (Table 3)

	LL-Ala	LD-Ala	LL-Phg	LD-Phg
**Formula**	$C_6H_{10}N_2O_2$	$C_6H_{10}N_2O_2$	$C_{16}H_{14}N_2O_2$	$C_{16}H_{14}N_2O_2$
**Crystal system**	Triclinic	Monoclinic	Triclinic	Orthorhombic
**Space group**	*P1*	$P2_1/n$	*P1*	*Pbca*
**a (Å)**	5.1552(4)	6.3497(3)	6.221(2)	10.127(9)
**b (Å)**	8.0596(5)	6.2203(4)	10.109(3)	8.205(7)
**c (Å)**	4.6698(2)	9.0438(5)	10.929(5)	15.578(12)
**α (°)**	103.16	90	81.20	90
**β (°)**	103.68	95.81	86.78	90
**γ (°)**	97.59	90	77.74	90
**Volume (Å^3)**	179.99	355.368	663.52	1294.53
**Z/Z′**	1/1	2/0.5	2/1	4/0.5
**ρ_{calc} (g/cm^3)**	1.312	1.329	1.333	1.366
**Temp. (K)**	293	293	293	293
**R_1**	3	3.7	6.52	3.83
**Ref. code**	LCDMPP01	TRDMPP01	-	GANJIQ
**CCDC No.**	-	-	1039283	790250

13. List of hydrogen bonds and related parameters in crystals of CDPs (Table 4)

CDPs	Donor−H···Acceptor	H···A (Å)	D···A (Å)	∠D−H···A (deg)
**LL-Ala**	N1−H1···O2	1.90	2.89	169
	N2−H2···O1	1.88	2.88	171
	C5−H7···O2	2.40	3.32	141
**LD-Ala**	N1−H1···O1	1.93	2.89	157
	C2−H2···O1	2.41	3.43	156
**LL-Phg**	N1−H13···O2	1.87	2.86	172
	N2−H14···O1	1.87	2.88	173
	C10−H10···O2	2.48	3.32	134
	C15−H15···O1	2.42	3.20	128
**LD-Phg**	N1−H7···O1	2.00	2.95	155
	C4−H1···O1	2.37	3.34	148

14. References

1 Kiran, M. S. R. N., Varughese, S., Reddy, C. M., Ramamurty, U. & Desiraju, G. R. Mechanical Anisotropy in Crystalline Saccharin: Nanoindentation Studies. *Cryst. Growth Des.* **10**, 4650-4655 (2010).

2 Varughese, S. *et al.* Interaction anisotropy and shear instability of aspirin polymorphs established by nanoindentation. *Chem. Sci.* **2**, 2236-2242 (2011).

3 Varughese, S., Kiran, M. S. R. N., Ramamurty, U. & Desiraju, G. R. Nanoindentation as a Probe for Mechanically-Induced Molecular Migration in Layered Organic Donor–Acceptor Complexes. *Chem. Asian J.* **7**, 2118-2125 (2012).

4 Ghosh, S. & Reddy, C. M. Elastic and Bendable Caffeine Cocrystals: Implications for the Design of Flexible Organic Materials. *Angew. Chem. Int. Ed.* **51**, 10319-10323 (2012).

5 Krishna, G. R., Kiran, M. S. R. N., Fraser, C. L., Ramamurty, U. & Reddy, C. M. The Relationship of Solid-State Plasticity to Mechanochromic Luminescence in Difluoroboron Avobenzone Polymorphs. *Adv. Funct. Mater.* **23**, 1422-1430 (2013).

6 Sahoo, S. C. *et al.* Kinematic and Mechanical Profile of the Self-Actuation of Thermosalient Crystal Twins of 1,2,4,5-Tetrabromobenzene: A Molecular Crystalline Analogue of a Bimetallic Strip. *J. Am. Chem. Soc.* **135**, 13843-13850 (2013).

7 Mishra, M. K., Sanphui, P., Ramamurty, U. & Desiraju, G. R. Solubility-Hardness Correlation in Molecular Crystals: Curcumin and Sulfathiazole Polymorphs. *Cryst. Growth Des.* **14**, 3054-3061 (2014).

8 Chattoraj, S. *et al.* Origin of Deteriorated Crystal Plasticity and Compaction Properties of a 1:1 Cocrystal between Piroxicam and Saccharin. *Cryst. Growth Des.* **14**, 3864-3874 (2014).

9 Mishra, M. K., Desiraju, G. R., Ramamurty, U. & Bond, A. D. Studying Microstructure in Molecular Crystals With Nanoindentation: Intergrowth Polymorphism in Felodipine. *Angew. Chem. Int. Ed.* **53**, 13102-13105 (2014).

10 Mishra, M. K., Varughese, S., Ramamurty, U. & Desiraju, G. R. Odd–Even Effect in the Elastic Modulii of α,ω-Alkanedicarboxylic Acids. *J. Am. Chem. Soc.* **135**, 8121-8124 (2013).

11 Omenetto, F. G. & Kaplan, D. L. New Opportunities for an Ancient Material. *Science* **329**, 528-531 (2010).

12 Meyers, M. A., Chen, P.-Y., Lin, A. Y.-M. & Seki, Y. Biological materials: Structure and mechanical properties. *Prog. Mater. Sci.* **53**, 1-206 (2008).

13 Ebenstein, D. M. & Pruitt, L. A. Nanoindentation of biological materials. *Nano Today* **1**, 26-33 (2006).

14 Chen, W. F. & Lui, E. M. *Handbook of Structural Engineering* (CRC Press, 2005).

15 Yoda, M. *et al.* Bond strength of binary titanium alloys to porcelain. *Biomaterials* **22**, 1675-1681 (2001).
